# Multi-parameter systematic strategies for predictive, preventive and personalised medicine in cancer

**DOI:** 10.1186/1878-5085-4-2

**Published:** 2013-01-22

**Authors:** Rong Hu, Xiaowei Wang, Xianquan Zhan

**Affiliations:** 1 Key Laboratory of Cancer Proteomics of Chinese Ministry of Health, Xiangya Hospital, Central South University, 87 Xiangya Road, Changsha, Hunan, 410008, People’s Republic of China; 2 Hunan Engineering Laboratory for Structural Biology and Drug Design, Xiangya Hospital, Central South University, 87 Xiangya Road, Changsha, Hunan, 410008, People’s Republic of China; 3 State Local Joint Engineering Laboratory for Anticancer Drugs, Xiangya Hospital, Central South University, 87 Xiangya Road, Changsha, Hunan, 410008, People’s Republic of China

**Keywords:** Predictive, preventive and personalised medicine, Genomics, Transcriptomics, Proteomics, Metabolomics, Systems biology, Multi-parameter systematic strategy

## Abstract

Cancer is a complex disease that causes the alterations in the levels of gene, RNA, protein and metabolite. With the development of genomics, transcriptomics, proteomics and metabolomic techniques, the characterisation of key mutations and molecular pathways responsible for tumour progression has led to the identification of a large number of potential targets. The increasing understanding of molecular carcinogenesis has begun to change paradigms in oncology from traditional single-factor strategy to multi-parameter systematic strategy. The therapeutic model of cancer has changed from adopting the general radiotherapy and chemotherapy to personalised strategy. The development of predictive, preventive and personalised medicine (PPPM) will allow prediction of response with substantially increased accuracy, stratification of particular patient groups and eventual personalisation of medicine. The PPPM will change the approach to tumour diseases from a systematic and comprehensive point of view in the future. Patients will be treated according to the specific molecular profiles that are found in the individual tumour tissue and preferentially with targeted substances, if available.

## Review

### Introduction

Cancer is a complex disease that is caused by the interplay of multiple internal factors and extrinsic factors [1,2]. DNA binding and induction of mutations in cancer-susceptibility genes are considerable mechanisms of tumour initiation. It is also reported that over 80% of cancer deaths in Western industrial countries can be attributed to extrinsic factors such as tobacco, alcohol, diet, infections and occupational exposures [1].

Alterations in multiple genes expression in cancer lead to dysregulation of the normal cellular programme for cell division, differentiation, apoptosis and proliferation. This results in an imbalance of cell replication and cell death, which favours growth of a tumour cell population [3,4]. Clinically, cancer appears to be many different diseases with different phenotypic characteristics. As cancer progresses, the genetic drift in the cell population produces cell heterogeneity with characteristics including cell antigenicity, invasiveness, metastatic potential, rate of cell proliferation, differentiation state and response to chemotherapeutic agents [5,6].

In the past 10 years, a number of radiotherapy and chemotherapy strategies available to treat cancer have increased [7]. Much of medical practice is based on standards of care. These interventions are based on knowledge and experience from the different levels of evidence generated by epidemiological and clinical studies or evidence-based medicine [8,9]. However, large randomised studies are designed to determine approach for the average populations, but not for specific individuals, which results in the therapeutic model ‘the same therapeutic strategy for the same type of disease’. In addition, basic scientists and clinicians have raised a lot of fundamental questions regarding identification of the causes of cancer, preventive measurement taken, the common characters in tumour susceptible group, predictive measurement used, the basic mechanisms of malignant transformation of cells, possibility of the use of the gene expression patterns of cancer cells to identify targets for cancer diagnosis or therapy, the suitability of the use of the common single parameter treatment measurement for all tumour patients, and the necessary or appropriate type of clinical trials for personalised therapeutic modality. Based on these questions, we propose the use of the multi-parameter systematic strategy to predict, prevent and personalise the treatment of a cancer. The multi-parameter systematic strategy for predictive, preventive and personalised medicine (PPPM) in cancer was initially conceived by the Zhan and Desiderio [10]; this concept was addressed by XZ as a keynote speaker and panellist at the first EPMA-World Congress 2011 and was collected into the post-meeting report of the first EPMA-World Congress 2011 (or called “EPMA White Paper”) [11].

### Pathophysiological basis of multi-parameter systematic strategies for PPPM in cancer

From a clinical point of view, cancer is a large group of diseases that vary in their age of onset, rate of cell proliferation, state of cellular differentiation, invasiveness, metastatic potential, diagnostic detectability, response to treatment and prognosis [12-14]. From a molecular biological point of view, cancer is a kind of gene disease and results in a series of molecular changes, which is correlated with signal transduction system, cell cycle, differentiation and apoptosis [15,16].

Not only one intracellular signal pathway is involved in the molecular mechanisms of a cancer [17]. For example, several research groups have demonstrated that phosphoinositide 3-kinase (PI3K/Akt), mitogen-activated protein kinase (MAPK) and signal transducer, and activator of transcription 3 (STAT3) pathways were activated in obesity-associated colon cancer. Mammalian target of rapamycin (mTOR), a down-stream of both PI3K/Akt and MAPK, is highly activated [18]. Activated mTOR in turn inhibits the PI3K/Akt pathway and further activates the STAT3 pathway [19]. Elucidation of multiple signal pathways has therapeutic implications. The activity of PI3K/Akt may increase significantly if mTOR is inhibited because of the feedback inhibition of mTOR on PI3K activity [20]. Thus, it is highly important to simultaneously inhibit both mTOR and PI3K in the treatment of obesity-associated cancer. Thus, a number of small molecules which both inhibit PI3K and mTOR have been developed [21]. They include BEZ-235, SF1126 and XL765, which are more effective than single inhibitors of PI3K or mTOR in cancer therapy. SF1126 and XL765 has been used for phase I clinical trials, and BEZ-235 has been used in phase II clinical trials in the treatment of several cancers [22-26]. Thereby, traditional investigation focusing on single-molecule biomarker or target in tissue or plasma for cancer prediction and prevention is an unrealistic assumption. Not one single-parameter can resolve an entire problem, or sometimes, one parameter is unable to resolve a problem at all. Multiple inhibitors could provide a novel approach to inactivate signal pathways and are likely to have a better therapeutic effect than any one single-inhibitor.

Tumour heterogeneity is another important character of malignant tumour [10,27-30]. Heterogeneity is observed in very part of the tumour, not only in the same tumour among different patients, but also in all tumour progression stages of the same individual patients [31]. The genetic instability of tumour cell is an important factor of tumour progression and heterogeneity, and results in somatic mutation which appears as an increasing phenotypic variability of the tumour cell group [32,33]. However, most treatment schemes were designed according to the doctor’s experience. The therapeutic model ‘the same therapeutic strategy for the same type of disease’ commonly existed so its curative effect cannot achieve the expected purpose. Heterogeneity was often ignored in tumour treatment. Tumour cell genetic instability often appears many cell subsets with different biology characteristics [34,35]. These cells subsets have different sensitivity to various therapeutic agents [36]. Some tumour gene expression resulted in the treatment resistance. Moreover, the treatment factor itself (such as radiotherapy and chemotherapy) is also a mutagen that promotes formation of new cell subsets in tumour progression especially when the treatment is not suitable. Obviously, tumour heterogeneity has greatly trapped the tumour treatment. On the other hand, the individual differences between tumour patients, such as the function of liver and kidney, age, physical condition and personal lifestyle factors, are also another important factor which impacts on the tumour treatment [37].

**Figure 1:**
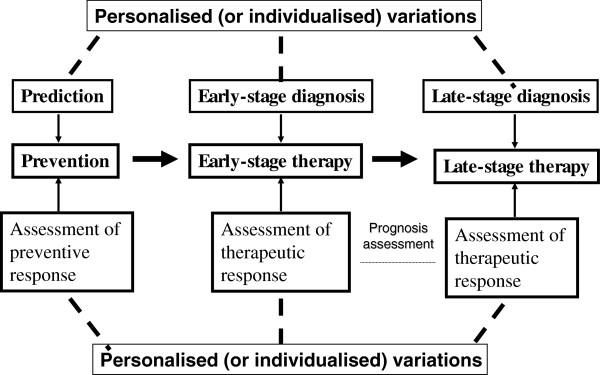
Personalised (or individualised) variations are involved in each aspect of healthcare.

Tumour heterogeneity and individual difference are actually derived from individualised (or personalised) variations. No two completely same individuals exist in the world. Variations are involved in each aspect of healthcare (Figure 1). Human healthcare includes three main stages: prediction/prevention, early stage diagnosis/early-stage therapy and late-stage diagnosis/late stage therapy. The assessment of preventive response will measure the efficacy of preventive intervention. The assessment of therapeutic response, namely, prognostic assessment, will measure the efficacy of therapeutic intervention. Towards the goal of human health, the prediction/prevention is the most important stage among the three stages because it will keep one in the status of no disease. Early stage diagnosis/therapy is the better strategy to halt the development of cancer when preventive intervention failed. Currently, most molecular medical studies focus on the discovery of biomarker towards the goal of prediction/prevention and early stage diagnosis/therapy. Late stage diagnosis/therapy is commonly called clinical diagnosis and treatment of a cancer. However, even though during the late stage diagnosis/therapy, variations are still involved in this stage; for example, radiotherapy and chemotherapy will vary among individuals.

In summary, cancer is a complex disease and can be initiated by various factors [1,2]. Tumour progression is accompanied by multitude of changes in metabolisms and cell signal pathways [17]. Tumour heterogeneity and individual differences, or called individualised variations, also hamper cancer healthcare. It is difficult for one to use traditional single-factor strategy to perform PPPM practice in cancer healthcare. Thereby, these factors determine the necessity of the multi-parameter systematic strategy for PPPM in cancer.

### Novel strategies and technologies for multi-parameter systematic strategies for PPPM in cancer

Chemotherapy and radiotherapy are the main therapeutic schemes for cancer in clinic [38,39]. The major obstacles of cancer chemotherapy are the development of drug resistance and the severe side effects [40]. Due to the modest tumour specificity of many anticancer drugs, normal tissues are also damaged. This prevents the application of sufficient high doses to eradicate less sensitive tumour cell populations. Thereby, tumours develop drug resistance that leads to treatment failure and fatal consequences for patients. Cancer radiotherapy also appears to develop radiation resistance and severe side effects [41] in patients. The efficacy of chemotherapy and radiotherapy of cancer extensively varies among individuals. Moreover, the prediction/prevention and early stage diagnosis/therapy in the molecular levels of cancer remain important and difficult issues. In addition, individualised variations are involved in each stages of cancer healthcare, which leads to the complexity of PPPM in cancer. Novel strategies and technologies such as ‘omics’ and systems biological techniques would be of great benefit for PPPM in cancer.

#### Genomics

Recent genomics technical breakthroughs have facilitated sequencing of the whole genome, genome-wide methylation analysis and transcriptome arrays in individual patients [42,43]. High-throughput sequencing now allows one to sequence more numbers of study patients and will facilitate for a therapeutic decision [42-44]. Previous high-throughput studies on gene expression profiling and epigenetic alterations in tumour cells have generated large amounts of data. The complexity of these data may result from the heterogeneity of cell material that will provide an averaging of results among tumour cells, stroma, endothelium and blood cells, and lead to longstanding genomic instability [45,46]. In turn, DNA studies, while more definitive in the presence of clonal tumour cells, have been hampered by the inability to readily detect unknown lesions; polymerase chain reaction amplification technologies can usually only arrest known suspects but fail to identify culprits among the general population of genes that have not been incriminated by previous evidence. Another weakness is the lack of a proper reference to the same individual’s normal tissues. Yet truly predictive or informative studies have proven significantly more difficult than originally anticipated.

#### Transcriptomics

Transcriptomics has been used to describe the global mRNA expression of a particular tissue, yielding information about the transcriptional differences between two or more states [28,47,48]. Understanding the transcriptomics is essential for interpreting the functional elements of the genome and revealing the molecular constituents of cells and tissues and also for understanding cancer development.

Microarray allows analysis and quantification of the entire transcriptome profile of an organism [49]. Hybridisation-based approaches typically involve incubating fluorescently labelled cDNA with custom-made microarrays or commercial high-density oligonucleotide microarrays. Specialised microarrays have also been designed; for example, arrays with probes spanning exon junctions can be used to detect and quantify distinct spliced isoforms [50]. Genomic tiling microarrays have been constructed and allowed the mapping of transcribed regions to a very high resolution, from several base pairs to approximately 100 bp [51,52]. Hybridisation-based approaches are high throughput and relatively inexpensive. A vast literature has accumulated on the use of microarray profiling in cancer diagnosis and classification. For example, DNA microarray gene expression profiling can detect lymph node metastases for primary head and neck squamous cell carcinomas [53]. Combining transcriptional and metabolic data from the same breast carcinoma sample contributes to a more refined subclassification of breast cancers as well as reveals relations between metabolic and transcriptional levels [54]. Thus, microarray technology is a powerful resource for transcriptomes development in cancer.

However, these methods have several limitations, which include reliance upon existing knowledge about genome sequence, high background levels owing to cross-hybridisation [55,56] and a limited dynamic range of detection owing to both background and saturation of signals. Moreover, comparing expression levels across different experiments is often difficult and can require complicated normalisation methods.

#### Proteomics

Proteomics mainly applies to the identification, characterisation and quantitation of the protein in a defined system (organelle, cell, tissue, biofluid or whole organisms) [57-59]. Due to its ability to detect a large number of proteins in a short period of time, proteomics has been considered as a powerful tool for the study of human tumour [60]. The commonly used quantitative proteomic methodologies are (1) gel-based, two-dimensional polyacrylamide gel electrophoresis [28,57,61,62] and two-dimensional difference in-gel electrophoresis [63,64] and (2) ‘gel-free’ isotope-tagging/labelling technologies, including isotope-coded affinity tagging [65,66], stable isotope labelling with amino acids in cell culture [67], proteolytic ^18^O labelling [68,69] and stable isotope-tagged amine-reactive reagents (iTRAQ) [70-73] and more recently, “label-free” mass spectrometry (MS)-based proteomics [74,75]. In general, gel-free methods can address many of the shortcomings of gel-based approaches, which is tedious and inefficient in resolving proteins that are lowly abundant, insoluble or large (>200 kDa) [57]. Gel-free methods have rapidly been incorporated in proteomic laboratories, since they are able to overcome many of the limitations of gel-based approaches [57,76]. These main technological tools are typically followed by identification of interest spots using MS analysis which allows highly sensitive and high-throughput identification of proteins/peptides and post-translational modifications. Briefly, a large variation of MS technologies is currently available, which evolved from electrospray ionisation (ESI) and matrix-assisted laser desorption/ionisation (MALDI) to a new generation of mass analysers, such as hybrid quadrupole time-of-flight (TOF) and tandem time-of-flight (TOF/TOF) instruments [77,78]. While newer and higher capacity MS technologies, such as LTQ-FT-MS and orbitrap type of analysers, are being developed [79,80], the most commonly used MS technologies include MALDI-TOF, SELDI-TOF and ESI-MS/MS.

By using these technologies, a lot of biomarkers and drug targets in cancer have been discovered. For example, Zhan et al. have identified proteins, including pituitary hormones, cellular signals, enzymes, cellular-defence proteins, cell structure proteins and transport proteins in human pituitary adenoma using 2-DE with MALDI-TOF and liquid chromatography-electrospray ionisation-quadrupole ion trap (LC-ESI-Q-IT) [28,57,81,82]. The phosphorylation sites at Ser-77 and Ser-176 of human growth hormone were also found in the normal (control) pituitary and in adenomas [83]. Secretagogin was found to be a potential biomarker for human nonfunctional pituitary adenoma [57,82]. GSTP1, HSPB1 and CKB were found to be novel potential biomarkers for early detection of lung squamous cell carcinoma by using iTRAQ-tagging combined with two-dimensional liquid chromatography tandem MS analysis, while GSTP1 down-regulation is involved in human bronchial epithelial carcinogenesis [84]. The protein profiles in response to epidermal growth factor (EGF) treatment in ovarian cancer cells were analysed using iTRAQ labelling and mass spectrometry [85].One of the differentially regulated proteins, lysosomal-associated membrane protein-1, in different stages of epithelial ovarian cancers was found to be a promising biomarker in understanding the progression of EGF-stimulated ovarian cancers and to be useful in the prediction of treatment responses involving tyrosine kinase inhibitors or EGF receptor monoclonal antibodies [85].

It is also evident that current proteomic technologies are biased towards high-abundant proteins. A current challenge is, thus, the study of low-abundant proteins [57]. Because many proteins do perform their biological activity as a partner of protein groups, another area receiving increasing attention is the protein-protein interaction analysis and cell signal pathway. The identification of protein-protein interactions is important to understand the mechanisms of signal transduction and establishing intracellular signalling networks [86,87].

#### Metabolomics

Metabolomics is useful to predict the effect of metabolic pathways on anticancer drugs in tumours and patients [88]. By means of metabolomic techniques, global sets of low-molecular weight metabolites are measured as indicators of physiological or pathological states. Large-scale data obtained by metabolomic methods may contribute to construct molecular interaction and gene regulatory networks that are able to predict drug effects [89,90]. The development of drug resistance and severe side effects leads to treatment failure and fatal consequences for patients. Novel strategies to broaden the narrow therapeutic range by separating the effective dose and toxic dose would be of great benefit for the improvement of cancer chemotherapy.

The potential of ‘omics’ technologies for the pretherapeutic screening of markers may help to identify the best-tolerated and most effective treatment strategy at optimal dose scheduling according to individual ‘omics’ fingerprints of each cancer and each patient.

#### Systems biology

The human genome project has catalysed two major paradigm changes of systems biology and PPPM [91-93], which dominate the twenty-first century biology and medicine [94]. Life is a very complicated and systematic phenomenon, which is not inherent in DNA, RNA, proteins, carbohydrates or lipids but is a consequence of their actions and interactions [95]. Only analysis of one factor among them would not really reveal the essentiality of life and disease, which results in the difficulty in fighting against disease. Thus, elucidation of their actions and interactions must be necessary, which catalyses the coming out of systems biology. Systems biology is a comprehensive analysis of all the components of a biological system at a given conditions, which needs an interdisciplinary team of investigators who are also capable of developing high-throughput technologies and computational tools. The rapid development of ‘omics’ technologies, bioinformatics and computation biology accelerates the development of systems biology, which, in turn, revolutionises the traditional biology [95]. However, many technical challenges remain for systems biology, including (a) data quality and standardisation of ‘omics’-based large-scale data, (b) the immaturity of network biology, (c) the requirement of high-sensitivity tools for detection and quantification of the concentrations, fluxes and interactions of various types of molecules at a given space and time, (d) the necessity of miniaturised and automated microfluidics/nanotechnology platforms that are capable of multi-parameter analyses of cell sorting and single cell gene and protein profiling, (e) the need of imaging technologies that enable the dynamic, spatial and multi-parameter measurements within single cells, and (f) even challenges regarding fair credit and data ownership [95]. Systems biology will be much improved with resolving those challenges, which offers the great promise for the revolution in the medicine practice [96]. Under the concept of systems biology, the integration of ‘omics’ data, specific genetic traits and multi-parameter diagnostics techniques will improve and form novel assessing procedures of health and disease status, evolute the predictive and preventive medicine and restrict personalised medicine.

The rapid development and application of systems biology in disease is reforming basic medical research and clinical practice. In the future, medical researchers, clinicians and patients will be equipped with a deluge of personal information such as whole genome sequences, transcriptomic and proteomic profiling of diseased tissues, interactome network and periodic multi-analyte blood testing of biomarker panels for disease and wellness [96]. This information will enable accurate prediction and early diagnosis for a disease and personalised treatment of a patient. Of course, ones are still at the dawn of PPPM, the full implementation of which requires integration of basic and clinical researches through advanced systems thinking and the employment of high-throughput technologies in genomics, proteomics, nanofluidics, single-cell analysis and computation strategies.

**Figure 2:**
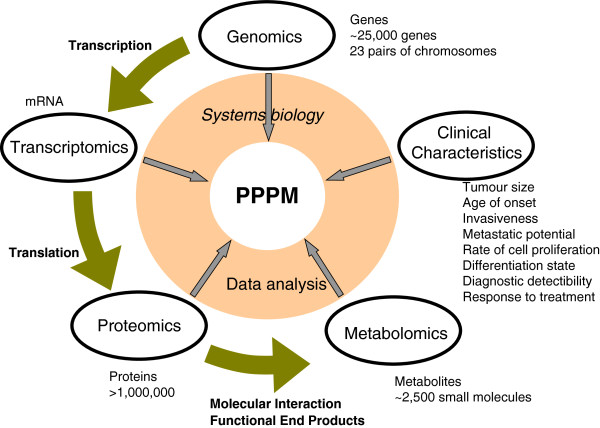
The contributions of ‘omics’ and systems biology to the practice of PPPM.

In summary, the development of ‘omics’ and systems biology strategies and techniques catalysed one to consider healthcare in a multi-parameter systematic angle. The ‘omics’ produces high-throughput data at the genome (DNA), transcriptome (mRNA), proteome (protein) and metabolomics (metabolite) levels. Systems biology can systematically integrate those ‘omics’ data to form a panel change of genes, proteins, metabolites and clinical features to predict, prevent and personalise the treatment of cancer (Figure 2). However, the question also arises as to which particular cytostatic agent and which combination of substances is most suited for an individual tumour. The concept of individualised therapy itself traces back to the 1950s [97]. The statistical probability of therapeutic success is well known for larger groups of patients from clinical therapy trials. However, it is not possible to predict how an individual tumour will respond to chemotherapy. Although clinic-pathological prognostic factors such as tumour size, lymph node and far distance metastases are valuable for the determination of prognosis of larger cohorts, those are less helpful for the development of personalised therapy options. With the current progress in molecular biology, the practice of PPPM in the clinic needs a lot of work to do.

**Figure 3:**
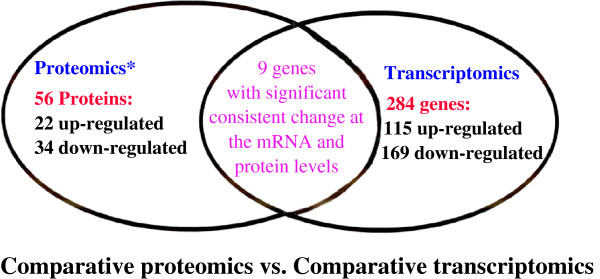
**Analysis of comparative proteomics vs. comparative transcriptomics in human nonfunctional pituitary adenomas.** *Comparative proteomics found 251 differential gel-spots, 93 differential gel-spots were excised for MS characterisation, and 56 proteins were identified.

**Figure 4:**
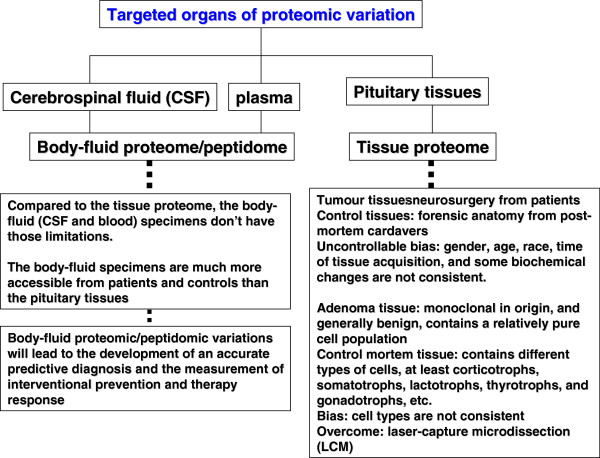
Targeted organs that are used to analyse proteomic variations in human nonfunctional pituitary adenomas.

**Figure 5:**
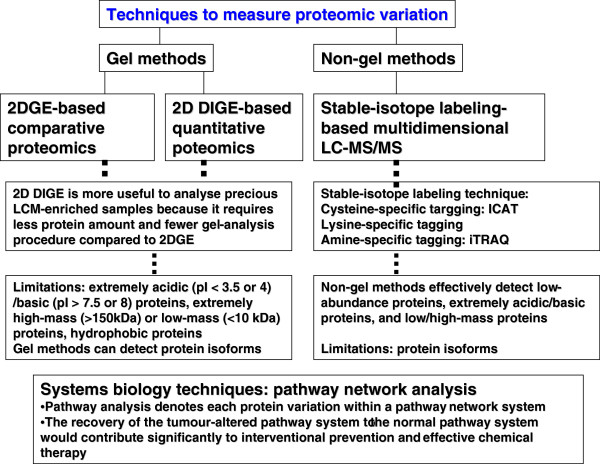
Techniques that are used to measure proteomic variations.

**Figure 6:**
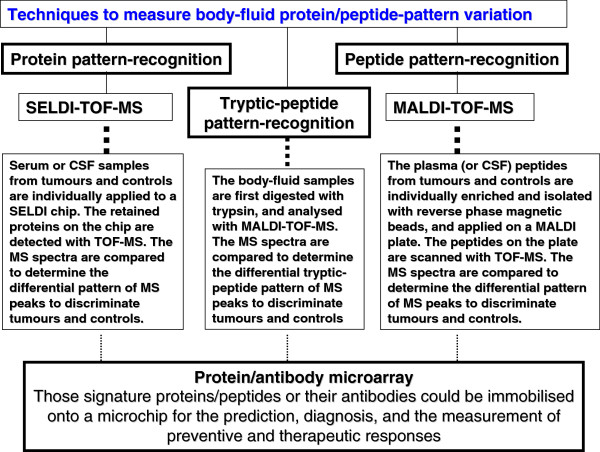
Techniques that are used to measure body fluid protein/peptide pattern variation.

Here, an example is taken regarding the use of proteomic and transcriptomic variations for PPPM in human nonfunctional pituitary adenoma. Proteomics and transcriptomics studies were performed in human pituitary adenoma [28,57,82]. A total of 56 differentially expressed proteins and 284 differentially expressed genes were identified in human nonfunctional pituitary adenomas compared to control pituitaries (Figure 3) [28,57,82]. Only nine genes were found with significant consistent changes at the mRNA and protein levels (Figure 3). A phenomenon was observed that at the gene level, a gene would not have a significant change, but at the protein level, its corresponding protein has a significant change. Also, at the gene level, a gene would have a significant change, but at the protein level, its corresponding protein had an indistinctive change. It indicates that the phenotype of a gene or protein was regulated in the complex genome and proteome system. The assumption based on the traditional single gene and protein as a biomarker for a cancer healthcare is no reality. Actually, it is very difficult for one to say that any single cancer-specific gene and protein is found. But it would be confident for one to say that a panel of genes/proteins was discovered to be related to human pituitary adenoma. It is very dangerous for those researchers who assume that the research failed without the discovery of single specific gene and protein. The reality is that there are a huge number of scientific publications regarding a single gene and protein, and each publication emphasises that its studied gene and protein are such important and specific to a disease. However, in regard to how much of those so-called specific gene and protein products contributed to the resolution of a disease in the disease system could be a doubt. Thus, from the angle of systems biology, four significant signalling pathway network variations that were associated with nonfunctional pituitary adenomas were discovered including mitochondrial dysfunction, oxidative stress, cell-cycle dysregulation and the MAPK-signalling abnormality [10,17]. Therefore, we propose that the use of a panel of genes/proteins as the biomarker would be more reliable and significant for prediction, prevention and personalised treatment of cancer. Based on the concept of multi-parameter systematic strategy, first, one should consider the human pituitary adenoma as a whole body disease then the targeted organs for proteomic variation analyses should include not only pituitary tissues, but also body fluid (cerebrospinal fluid and plasma). Figure 4 shows the contributions of pituitary tissue proteomic variations and of body-fluid proteomic/peptidomic variations to pituitary adenoma. Second, no single technique is perfect to measure the omic variation; one should consider the use of multiple techniques to measure the proteomic variations and protein/peptide pattern variations. Figure 5 shows the use of gel and non-gel methods to measure the pituitary adenoma proteomic variations and the use of systems biology techniques to denote the protein variation in the network system. Figure 6 clearly shows the techniques of body fluid protein/peptide pattern recognition including the SELDI-TOF-MS-based protein pattern recognition, the MALDI-TOF-MS peptide pattern recognition and tryptic-peptide pattern recognition; also, protein/antibody microarray would be another useful technique for multi-parameter systematic strategy of PPPM in cancer.

### Prospective thoughts regarding multi-parameter systematic strategies for PPPM in cancer

Recent technological advances, combined with the development of bioinformatics and systems biology tools, allow us to better address biological questions combining omic approaches (i.e. genomics, transcriptomics, proteomics and metabolomics). The rapid developments in omic technologies allow for a systematic investigation of genes, proteins and metabolites occurring in a tumour. The search for individual genomic, transcriptomic, proteomic and metabolomic variations in tumour diseases will help ones to better characterise the disease in each patient. The researchers have done a lot of omic data collection in tumour patients. However, these data are now not available to become integrated into clinical practice.

Here, a lot of problems exist since most of the researches focus on single-molecule biomarker and a single compound used for detecting a disease in clinic; however, the reality is characterised by heterogeneity among patients [10]. Cancer is initiated by numerous factors and causes a range of different molecular changes. The pituitary adenoma is taken for example; a significant elevation of blood growth hormone and prolactin has been useful to predict and diagnose acromegaly and prolatinoma, respectively. However, a large number of acromegaly and prolactinoma patients are still detected at an advanced stage. For the nonfunctional pituitary adenomas, no blood hormone levels are elevated, almost all nonfunctional pituitary adenomas are diagnosed at a late stage, and no molecular indices measure the therapy response and prognosis. Multiple-parameter biomarkers from serum or cerebrospinal fluid (CSF) will resolve these problems in prediction, prevention and personalised treatment and assessment of a nonfunctional pituitary adenoma. Several promising multi-parameter strategies from serum or CSF proteomes and peptidomes might meet those requirements and include protein/peptide pattern diagnostics, protein microarrays and an antibody microarray as described previously.

Authors agree how important to study the structure and functions of a single protein/gene. However, personalised variations are involved in each aspect of healthcare as shown in Figure 1. For prediction, early stage diagnosis, and personalised treatment of a cancer, ones are encouraged to consider it through a multi-parameter, systemic angle of PPPM.

**Figure 7:**
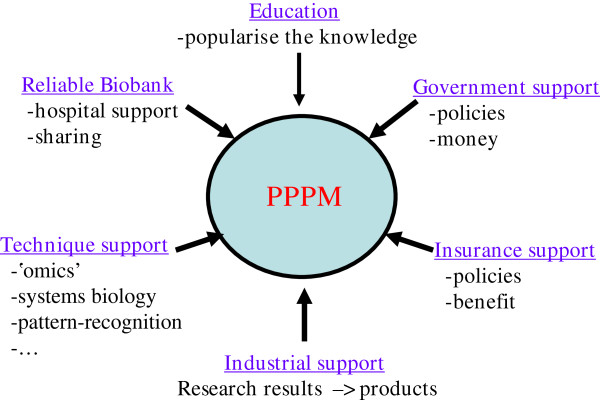
To realise PPPM is a systematic engineering and needs multiple supports.

Besides technical support such as the further development of a large amount of basic, clinical, bioinformatics and instrumental methods, the PPPM needs a lot of work to do (Figure 7): (a) a reliable biobank including standard sample collection method, standard cell separation method, authentic and detailed patient data; (b) government support such as policies and financial support; (c) education support which helps people know what is PPPM and accept it; (d) industrial support which transforms the research results to products and (e) insurance support including policies and benefit. Therefore, to realise PPPM is a systematic engineering and needs multiple pertinent supports. In addition, this comprehensive proposal needs to be supported by not only different individual research programmes but also international efforts such as the EPMA [11].

Recent findings have begun to improve analysis conditions in all of these areas, promising that the definition of new biomarkers will become much more precise. For RNA and protein characterisation, the choice of the proper phenotype to be analysed has begun to improve the feasibility of target identification in relevant cell populations. Enrichment technologies derived from other areas of stem cell research have allowed to highly enrich the small portion of true tumour-initiating cells capable of self-renewing growth and forming metastases from those cells that form the bulk of the tumour but never metastasise.

Two predictive markers (ER and HER2) for breast cancer are taken here for example. To date, these two markers have been established to predict efficacy of either endocrine or HER2-targeted therapy in breast cancer. The treatment of HER2-positive breast cancer is currently most advanced in terms of personalised breast cancer therapy. Besides the monoclonal antibody trastuzumab, further HER2-targeted drugs, namely tyrosine kinase inhibitors (TKIs) and other monoclonal antibodies, have been developed to overcome treatment resistance against trastuzumab. Lapatinib, a TKI against HER2 and HER1, disrupts the HER2 signalling pathway via inhibition of the intracellular tyrosine kinase activity. Lapatinib is active after trastuzumab failure and can enhance the efficacy of trastuzumab alone [98]. Further HER2-targeted agents such as pertuzumab or T-DM1 also have the same efficacy on breast cancer therapy.

Personalised treatment, however, does not mean to target all resistance mechanisms in all patients, but rather the identification of the right target agent or combination of agents for each individual patient. Thus, the comprehensive biomarker programmes to elucidate the specific mechanisms of resistance in individual patient are needed. Afterwards, an ever-growing list of new targeted agents, according to comprehensive biomarker, will be greatly effective for cancer therapy. The art of PPPM will be to select the right treatment for the right patient at the right time as opposed to the ‘one size fits all’ concept. In this respect, the integration of multiple omic results and procedures seems necessary. Therefore, an emerging challenge is the integration of the huge amount of data generated and the standardisation of the procedures and methods used. Functional data integration will lead to answers to unsolved questions and, hopefully, will be applicable to clinical practice and management of patients.

To establish PPPM for clinical practice, the efficient and fast bioinformatics and systems biology programmes are required to filter the relevant data useful for clinicians to guide their treatment decisions. Future cancer patients will receive a comprehensive genomic and proteomic analysis of their tumours that will allow oncologists to tailor therapies aimed at specific molecular lesions for maximum clinical benefit with minimal treatment-related toxicity. Not all of these findings will be useful for every patient; but step by step, knowledge of individual molecular lesions will lead to the development of surgical, radiological and medical approaches to provide an oncology patient with personalised medicine.

## Conclusions

The development of molecular biology and systems biology technologies to analyse DNA, RNA, protein and metabolite provides potential contributions to healthcare practice at both levels of local and holistic therapies of a tumour patient. These approaches have the potential to fulfill the promise of delivering the right dose for the right indication to the right patient at the right time. Importantly, PPPM offers the opportunity to increase therapeutic efficacy by targeting the genomic variations or proteomic variations driving tumour behaviour while, at the same time, decreasing inadvertent toxicity due to altered drug metabolism encoded by the patients’ genetic background. Besides molecular biology technologies development, the practice of PPPM also needs all aspects of support including education, government, reliable biobanks, industry and insurance. The traditional single-factor strategy should be shifted to the multi-parameter systematic strategy for PPPM in cancer.

## Abbreviations

CSF: Cerebrospinal fluid; EPMA: European Association for Predictive Preventive and Personalised Medicine; ESI: Electrospray ionisation; iTRAQ: Isobaric tags for relative and absolute quantification; LC: Liquid chromatography; MALDI: Matrix-assisted laser desorption/ionisation; MAPK: Mitogen-activated protein kinase; MS: Mass spectrometry; MS/MS: Tandem mass spectrometry; mTOR: Mammalian target of rapamycin; PI3K/Akt: Phosphoinositide 3-kinase; PPPM: Predictive, preventive and personalised medicine; Q-IT: Quadrupole ion trap; TOF: time-of-flight; STAT3: Signal transducer and activator of transcription 3; TOF/TOF: Tandem time-of-flight.

## Competing interests

The authors declare that they have no competing interests.

## Authors’ contributions

RH participated in the collection of references, constructed Figure 2 and drafted the most parts of the initial manuscript and participated in its partial revision. XW participated in the collection of references and drafted the section of systems biology. XZ conceived of the concept and idea of this article, participated in the collection of references, formed the thought line and outline of this article and constructed Figures 1, 3, 4, 5, 6 and 7 and their corresponding text descriptions. He also drafted the section that an example is taken regarding the use of proteomic and transcriptomic variations for PPPM in human nonfunctional pituitary adenomas as well as coordinated, guided, led and corresponded the writing and heavily revised the entire manuscript, and trained RH and XW regarding the concepts of PPPM and the multi-parameter systematic strategies for PPPM in cancer. All authors read and approved the final manuscript.

## Authors’ information

XZ is a professor of disease proteomics and structural biology at the State Local Joint Engineering Laboratory for Anticancer Drugs, Hunan Engineering Laboratory for Structural Biology and Drug Design, and Key Laboratory of Cancer Proteomics of Chinese Ministry of Health, Xiangya Hospital, Central South University, China. Before he returned to China in 2012, he had continuously worked in the United States of America for 11 years, where he achieved the rank of Associate Professor of Neurology at the University of Tennessee Health Science Center, USA. He is also a National Representative of EPMA in China, associate editor of BMC Genomics and BMC Medical Genomics, and Member of the Editorial Board of EPMA-Journal and Genomics Discovery. His main research interests are focused on disease proteomics and structural biology, biomarker and PPPM molecular targets. RH graduated from China Pharmaceutical University and achieved the Ph.D. degree in pharmacology in 2011; currently, she is working in Xiangya Hospital, Central South University, China, whose research interest is in cancer proteomics, structural biology and personalised medicine. XW is a graduate student of pathology and pathophysiology at the Xiangya Hospital, Central South University, who is supervised by Professor Xianquan Zhan and focuses on the studies of cancer proteomics, biomarker and structural biology.
